# Inhibition of Human Urokinase-Type Plasminogen Activator (uPA) Enzyme Activity and Receptor Binding by DNA Aptamers as Potential Therapeutics through Binding to the Different Forms of uPA

**DOI:** 10.3390/ijms23094890

**Published:** 2022-04-28

**Authors:** Nico Dreymann, Julia Wuensche, Wiebke Sabrowski, Anja Moeller, Denise Czepluch, Dana Vu Van, Susanne Fuessel, Marcus M. Menger

**Affiliations:** 1 Fraunhofer Institute for Cell Therapy and Immunology (IZI), Branch Bioanalytics and Bioprocesses (IZI-BB), D-14476 Potsdam, Germany; nico.dreymann@izi-bb.fraunhofer.de (N.D.); wiebke.sabrowski@izi-bb.fraunhofer.de (W.S.); anja.moeller@izi-bb.fraunhofer.de (A.M.); denise.czepluch@izi-bb.fraunhofer.de (D.C.); 2 Institute for Biochemistry and Biology, University of Potsdam, D-14476 Potsdam, Germany; 3 RiNA GmbH, D-12489 Berlin, Germany; julia@wundratsch.de; 4 Institute of Chemistry and Biochemistry-Biochemistry, Freie Universität Berlin, D-14195 Berlin, Germany; 5 Department of Urology, Technische Universität Dresden, D-01307 Dresden, Germany; dana.vuvan@uniklinikum-dresden.de (D.V.V.); susanne.fuessel@uniklinikum-dresden.de (S.F.)

**Keywords:** biomarker, cancer, cancer therapy, DNA aptamer, microscale thermophoresis (MST), SELEX, surface plasmon resonance spectroscopy (SPR), uPA, uPAR, urokinase

## Abstract

Urokinase-type plasminogen activator is widely discussed as a marker for cancer prognosis and diagnosis and as a target for cancer therapies. Together with its receptor, uPA plays an important role in tumorigenesis, tumor progression and metastasis. In the present study, systematic evolution of ligands by exponential enrichment (SELEX) was used to select single-stranded DNA aptamers targeting different forms of human uPA. Selected aptamers allowed the distinction between HMW-uPA and LMW-uPA, and therefore, presumably, have different binding regions. Here, uPAapt-02-FR showed highly affine binding with a K_D_ of 0.7 nM for HMW-uPA and 21 nM for LMW-uPA and was also able to bind to pro-uPA with a K_D_ of 14 nM. Furthermore, no cross-reactivity to mouse uPA or tissue-type plasminogen activator (tPA) was measured, demonstrating high specificity. Suppression of the catalytic activity of uPA and inhibition of uPAR-binding could be demonstrated through binding with different aptamers and several of their truncated variants. Since RNA aptamers are already known to inhibit uPA-uPAR binding and other pathological functions of the uPA system, these aptamers represent a novel, promising tool not only for detection of uPA but also for interfering with the pathological functions of the uPA system by additionally inhibiting uPA activity.

## 1. Introduction

Urokinase-type plasminogen activator (urokinase, uPA) is a serine protease that plays an important role in the fibrinolytic system by converting the inactive-form plasminogen into the catalytically active-form plasmin. Human uPA is produced and secreted as a single polypeptide, glycosylated zymogen pro-uPA, consisting of 411 amino acids with an N-terminal A-chain containing a growth factor domain (GFD, amino acids (aa) 1–49), a kringle domain (KD, aa 50–131) and a C-terminal B-chain containing the catalytically active serine protease domain (SPD, aa 159–411). Between the N-terminal A-chain and the C-terminal B-chain is a linker domain (aa 132–158) [1].

Due to different proteases, pro-uPA undergoes cleavage of the peptide bond between Lys158 and Ile159 to produce the 54 kDa catalytically active two-chain high-molecular-weight uPA (HMW-uPA) linked by a disulfide bond. HMW-uPA can be cleaved a second time in the linker sequence between Lys135 and Lys136 by various proteases into a catalytically active low-molecular-weight uPA of 33 kDa (LMW-uPA) with a serine protease domain and an inactive amino-terminal fragment (ATF) that contains the GFD and the KD. Binding of pro-uPA and HMW-uPA through the GFD to the glycolipid-anchored uPA receptor (uPAR) activates the conversion of plasminogen to plasmin on cell surfaces [1].

While pro-uPA has more limited enzymatic activity compared to the two-chain HMW-uPA, it is still able to induce plasmin activation, presumably through conformational change of pro-uPA via binding to uPAR [2]. The two-chain HMW-uPA that binds to uPAR is 250-fold more potent in converting plasminogen to plasmin and the main activator of plasmin leading to extracellular proteolysis [3]. This mediates several physiological and pathological pathways by activating growth factors and promatrix metalloproteases, degrading extracellular matrix components and basement membrane and allowing the formation of new matrix, which are all involved in various processes that are necessary for tumorigenesis and cancer progression [4]. In particular, pathophysiological mechanisms such as tumor angiogenesis, malignant cell proliferation, invasion of surrounding tissues, cell extravasation and tumor progression and metastasis are supported by uPA binding to its receptor uPAR [5].

Inhibition of the catalytic activity of uPA is naturally caused by the serine protease inhibitors plasminogen activator inhibitor-1 and-2 (PAI-1 and PAI-2). Here, PAI-1 is the major inhibitor, which makes the conversion to plasmin a highly controlled event. PAI-1 and uPA form a covalently linked complex, which is absorbed together with uPAR through endocytosis. This leads to degradation of the uPA-PAI-1 complex and releases free uPAR to the extracellular region [6].

Independent of proteolytic activity, the uPA-uPAR complex plays a key role in signal transduction by interacting with several adhesion molecules like integrins and vitronectin, cellular receptors and proteins of the extracellular matrix, to control cell proliferation, apoptosis, chemotaxis, adhesion and migration [7]. Several independent studies showed that uPA is widely discussed as a prognostic and diagnostic marker for human malignancies [8] since the elevated expression of uPA in many different cancer types is mainly correlated with a poor prognosis [5,9].

In addition to its diagnostic and prognostic value, uPA is a potential target for cancer therapies by direct inhibition of its proteolytic activity or by inhibition of the uPA-uPAR interaction, which has been shown to suppress tumor growth, tumor invasion and metastasis in animal models [10]. Blocking of the catalytic domain and thereby the catalytic activity was achieved by specific antibodies [11], overexpression of the endogenous inhibitors PAI-1 [12] and small-molecule inhibitors [13]. The serine protease inhibitor Mesupron (WX-671) resulted in reduced metastatic spread and an extended lifespan in clinical trials on pancreatic and breast cancer patients [14]. To inhibit the downstream actions of the uPA-uPAR complex, another strategy is to block binding of uPA to its receptor. Inhibition of the uPA-uPAR complex by human uPA-ATF as an antagonist of uPA-uPAR interaction might be a promising anti-invasion and anti-metastasis strategy by displacing uPA from its cognate receptor [15]. Urokinase-derived cyclic peptides that were synthesized similarly to the GFD of uPA that binds to uPAR showed promising results in hampering tumor growth and metastasis in animal models [16].

In addition to all these strategies, nucleic acid aptamers targeting proteases are suggested as a promising alternative not only for recognition but also as inhibitors of enzymatic and regulatory mechanisms [17,18]. Aptamers recognize their targets with high specificity and affinity corresponding to K_D_ values in the picomolar to low nanomolar range, and therefore, are comparable to antibodies. Several effective aptamers against proteases are known today [17,18]. Specifically for uPA, Dupont et al. [19] were the first to identify 2’-F-pyrimidine-modified RNA aptamers binding near to the GFD domain. These aptamers were able to block binding of uPA to its receptor uPAR with low nM IC_50_ values. Botkjaer et al. [20] were able to select 2‘-fluoro-pyrimidine-modified RNA aptamers binding to the catalytic domain of pro-uPA. The only DNA aptamers known today are reported for pro-uPA, where a pool of DNA aptamers was selected after 12 selection rounds and a similar group of sequences was revealed with increased affinity to pro-uPA compared to the initial library pool [21].

Here, we present novel, highly affine and specific DNA aptamers of up to 82-nt long that can bind to different forms of human uPA. Single-stranded DNA (ssDNA) aptamers were isolated by systematic evolution of ligands by exponential enrichment (SELEX) from a 42-nt random ssDNA-library with a theoretical diversity of 1.9 × 10^25^ different sequences. Binding of aptamers was characterized for its affinity and specificity by various assays, including a fluorescent dye-linked aptamer assay (FLAA), electrophoretic mobility shift assay (EMSA), surface plasmon resonance (SPR) spectroscopy and microscale thermophoresis (MST). Selected aptamers could discriminate between HMW- and LMW-uPA. Therefore, we assume that our aptamers bind either in the region of the amino-terminal fragment or the serine protease domain. We also found that aptamer binding inhibited the binding of uPA to its receptor uPAR and suppressed the catalytic activity of uPA. Given the worldwide increase in cancer, the early detection and therapy of cancer are among the most important tasks in today’s medicine. Due to the ease of synthesis and modification, selected aptamers represent interesting agents for basic research analysis and diagnostic applications besides other uPA-targeting agents like small molecules, peptides or antibodies. Due to the small size of aptamers, together with their polyanionic nature, which results in good tissue penetration and rapid blood clearance, they may also be promising agents for therapeutics, while showing no immunoreactivity and low-to-no toxicity [22]. Although previous research identified modified RNA aptamers that targeted uPA and inhibited binding of uPA to uPAR [19], to our best knowledge, no aptamers that can inhibit uPA activity have been reported to date. Therefore, our aptamers could be used for potential inhibition of both the functionally important interactions of uPA with its receptor and the catalytic activity of uPA in different types of cancer.

## 2. Results

### 2.1. Characterization of Isolated DNA Aptamers to Human Urokinase Revealed Binding Sequences by EMSA and FLAA

After 11 selection rounds for HMW-uPA and 12 selection rounds for LMW-uPA, enriched DNA pools were sequenced using NGS. Analysis of the sequences in the different SELEX pools after selection against HMW-uPA and LMW-uPA showed the number of enriched sequences in the respective DNA pool. After comparing and analyzing the data and sequences in each DNA pool, sequences that showed specific enrichment and were present in both DNA pools were selected to test them for binding to HMW-uPA and LMW-uPA by EMSA and FLAA. Sequences that showed binding against both uPA forms or only against HMW-uPA by EMSA and FLAA are reported in Table 1. Sequences are named as uPA aptamers: uPAapt-xx.

#### 2.1.1. Gel Shift Experiments (EMSA)

**Figure 1:**
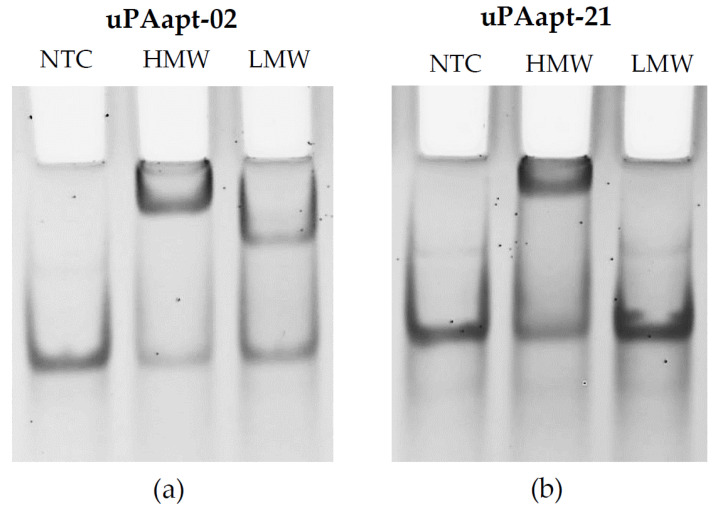
Binding of aptamers to HMW−uPA and LMW−uPA by EMSA. Gel shift experiments for (**a**) uPAapt−02 and (**b**) uPAapt−21. While uPAapt−02 demonstrated binding to both HMW−uPA and LMW−uPA, uPAapt−21 showed only binding to HMW−uPA when compared to the no-target control. NTC = no-target control, HMW = HMW−uPA, LMW = LMW−uPA.

Binding analysis for aptamers was qualitatively validated by gel shift experiments. Band shifting was observed for HMW-uPA with all aptamers, while band shifting for LMW-uPA was only observed for uPAapt-01, uPAapt-02 and uPAapt-03 compared to the no-target control (NTC). An example of band-shifting for uPAapt-02 and uPAapt-21 is shown in Figure 1. For detailed data on the gel shift experiment results for all full-length aptamers, see Table A1.

#### 2.1.2. Fluorescent-Dye Linked Aptamer Assay (FLAA)

**Figure 2:**
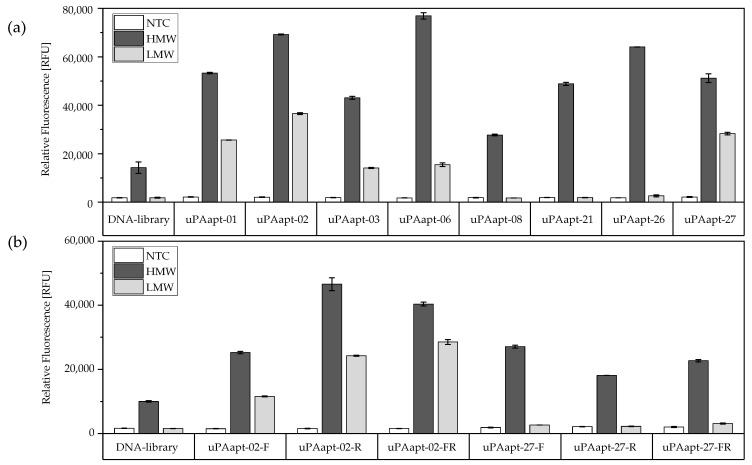
Binding of aptamers to HMW−uPA and LMW−uPA by FLAA. (**a**) Full−length−aptamers and (**b**) truncated versions of aptamers showed binding either to HMW−uPA or to both HMW−uPA and LMW−uPA when compared to their no-target control and the DNA-library. The relative fluorescence unit (RFU) for each sample is shown as the mean value of technical replicates. Error bars represent the range of measured values. NTC = no-target control, HMW = HMW−uPA, LMW = LMW−uPA. Number of records *n* = 2.

In FLAA experiments, the highly diverse ssDNA-library used as a control showed a low binding signal to HMW-uPA, which indicates slight, non-specific binding of DNA sequences. However, compared to the ssDNA-library and the no-target control, all aptamers showed higher signals, which indicates binding of aptamers to HMW-uPA. Binding to LMW-uPA was also identified for aptamers uPAapt-01, uPAapt-02, uPAapt-03, uPAapt-06 and uPAapt-27 by FLAA (Figure 2a). Additionally, several sequences were truncated by the forward-primer binding site (uPAapt-xx-F), reverse-primer binding site (uPAapt-xx-R) and both primer binding sites (uPAapt-xx-FR). Truncated aptamers were used to test whether primer binding regions are important to form the secondary structure binding uPA or whether the variable region alone is responsible for the uPA-binding structure. Binding to the different uPA-forms was still detected for truncated variants of aptamers uPAapt-02 and uPAapt-27. Here, high signals were detected for truncated versions of uPAapt-02 for HMW- and LMW-uPA and truncated variants of uPAapt-27 for HMW-uPA (Figure 2b).

### 2.2. Aptamers Showed High Affinity to uPA with up to pM K_D_ Values

**Figure 3:**
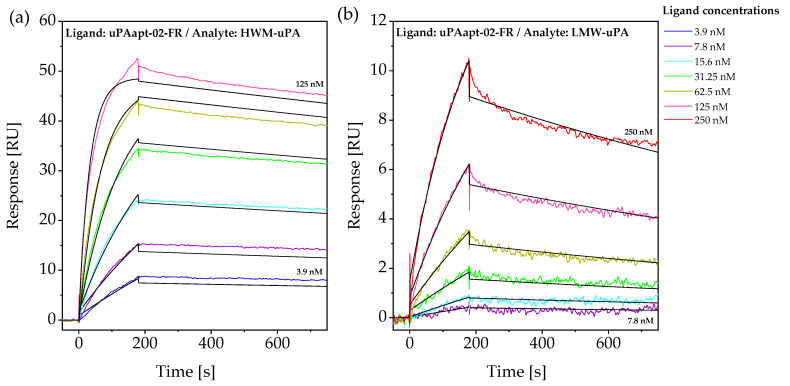
SPR sensograms of uPAapt−02−FR with (**a**) HMW−uPA and (**b**) LMW−uPA. The sensograms show each response unit (RU) over the time (s) of six different urokinase concentrations (for HMW−uPA, 3.9, 7.8, 15.6, 31.25, 62.5 and 125 nM, and for LMW−uPA, 7.8, 15.6, 31.25, 62.5, 125 and 250 nM) overlaid and globally fitted to a 1:1 binding model, to obtain values for the rate and equilibrium constants.

Subsequently, identified uPA-binding aptamers (Table 1) and several truncated variants were used for SPR experiments. Injection of uPA dilution series allowed for calculation of kinetic data such as the association and dissociation rates (k_a_ and k_d_) and resulting dissociation constants (K_D_s) of the binding event. For calculation, signals derived from the reference channel were subtracted. Obtained sensograms were fitted to a 1:1 Langmuir binding model using Biacore™ Evaluation Software (version 3.2, GE Healthcare Bio-Sciences AB, Uppsala, Sweden). While binding to HMW-uPA was detected for each aptamer with a K_D_ in a pM-nM range, binding to LMW-uPA could only be detected for aptamers uPAapt-01, uPAapt-02, uPAapt-02-R, uPAapt-02-FR and uPAapt-21 in an nM-range. While uPAapt-01 showed binding to LMW-uPA, no binding was detected for its truncated variant uPAapt-01-FR, which bound only to HMW-uPA. An example of fitted sensograms for binding of uPAapt-02-FR to HMW-uPA and LMW-uPA is shown in Figure 3.

For the binding of uPAapt-02-FR to HMW-uPA, the association rate was determined as 2.42 × 10^5^ M^−1^ s^−1^ and the dissociation rate was determined as 1.72 × 10^−4^ s^−1^. A K_D_ value of 7.08 × 10^−10^ M was obtained based on the kinetic data. For binding of uPAapt-02-FR to LMW-uPA, the k_a_ was 2.47 × 10^4^ M^−1^ s^−1^ and the k_d_ was 5.30 × 10^−4^ s^−1^. The calculated K_D_ value for binding to LMW-uPA was 2.14 × 10^−8^ M. The chi^2^, a standard statistical method that shows the closeness of a fit, was 1.80 for HMW-uPA and 0.02 for LMW-uPA. For detailed data of the on and off rates, including the chi^2^ and calculated dissociation constants of selected aptamers, see Table A2.

### 2.3. uPA Aptamer uPAapt-02-FR Demonstrated High Specificity for Human uPA

In addition to SPR, MST was used as an immobilization-free method to determine binding and associated K_D_ values when both binding partners are free in solution. Detection of binding events is enabled by one binding partner being fluorescently labeled. Here, uPAapt-02-FR was used as a representative for further characterization, as uPAapt-02-FR can bind both uPA forms with high binding affinities and K_D_ values in the low nM range. Besides binding to HMW- and LMW-uPA, cross-reactivity with pro-uPA, mouse uPA and tPA was tested for uPAapt-02-FR by MST (Table 2).

**Figure 4:**
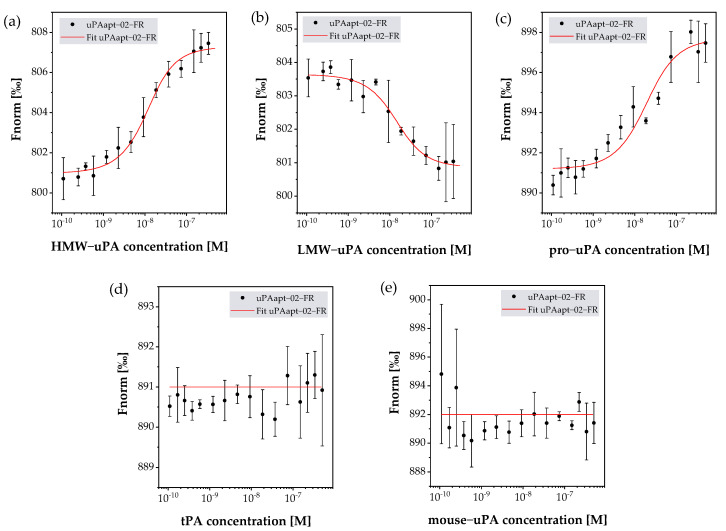
Binding analysis for uPAapt−02−FR with (**a**) HMW−uPA, (**b**) LMW−uPA, (**c**) pro−uPA, (**d**) tPA and (**e**) mouse−uPA using MST. MST measurements were carried out with 5′−Cy5−uPAapt−−02−FR and varying concentrations of the different targets (for HMW− and LMW−uPA, from 0.1 nM to 333 nM, and for pro−uPA, tPA and mouse−uPA, from 0.1 nM to 500 nM). Each plot shows the normalized fluorescence Fnorm (‰) vs. the concentration (M) of the given target. While uPAapt−02−FR showed binding curves for HMW−uPA, LMW−uPA and pro−uPA, no binding was detected for tPA or mouse−uPA. The Fnorm for each sample is shown as the mean value of independent experiments. Error bars represent the standard deviation. Red lines represent the fit of data points using the law of mass action. Number of records *n* = 3.

K_D_ values determined using MST were comparable to those calculated from SPR data with a K_D_ value of 6.69 × 10^−9^ M for HMW-uPA and 9.40 × 10^−9^ M for LMW-uPA. Furthermore, binding to pro-uPA was detected at a K_D_ value of 1.40 × 10^−8^ M. No binding, and therefore, no cross-reactivity was detected for uPAapt-02-FR with mouse uPA and tPA. Examples for binding and no-binding curves of uPAapt-02-FR are shown in Figure 4.

In addition, uPAapt-21 was also tested for binding to HMW- and LMW-uPA. Here, uPAapt-21 was used as a representative that binds only the HMW-uPA form since it showed binding only to HMW-uPA in EMSA and FLAA. However, in SPR, uPAapt-21 showed binding to both HMW-uPA and LMW-uPA (Table A2). Since, for this aptamer, some techniques showed binding only to HMW-uPA, while others showed binding to both HMW-uPA and LMW-uPA, MST was used to verify its LMW-uPA binding potential with another highly sensitive method. Here, binding of uPAapt-21 could be confirmed with a K_D_ value of 2.08 × 10^−7^ M for HMW-uPA and 1.41 × 10^−6^ M for LMW-uPA. Dissociation constants (K_D_s) including the K_D_ confidence of a binding event between uPAapt-02-FR or uPAapt-21 and used proteins are shown in Table 2.

### 2.4. Various Aptamers Reveal an Inhibitory Effect toward Binding of uPA to uPAR with up to nM IC_50_ Values

**Figure 5:**
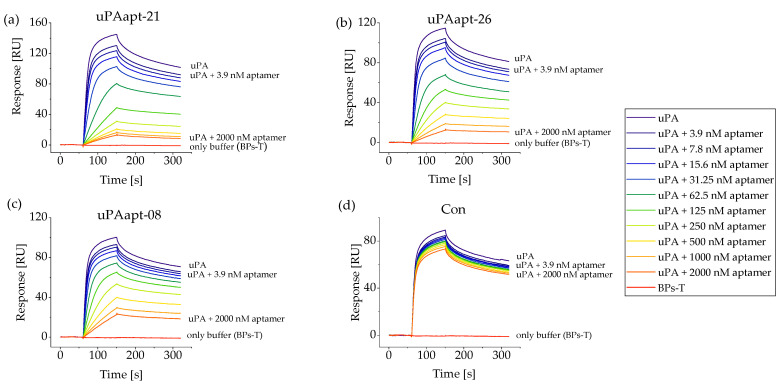
Inhibition of uPA binding to uPAR by selected aptamers. SPR sensograms show the capture of 20 nM human uPA (injected from 60 to 150 s) on a sensor surface with immobilized human uPAR in the presence of increasing concentrations of aptamers. Sensograms display each response unit (RU) over the time (s) for uPA alone or in complex with different aptamer concentrations (3.9, 7.8, 15.6, 31.3, 62.5, 125, 250, 500, 1000 and 2000 nM). (**a**) uPAapt−21, (**b**) uPAapt−26, (**c**) uPAapt−08 and (**d**) Con. While uPAapt−21, uPAapt−26 and uPAapt−08 showed a dose-dependent effect on the inhibition of uPA binding to uPAR, the sequence-unrelated control aptamer Con showed no inhibitory effect.

**Figure A1:**
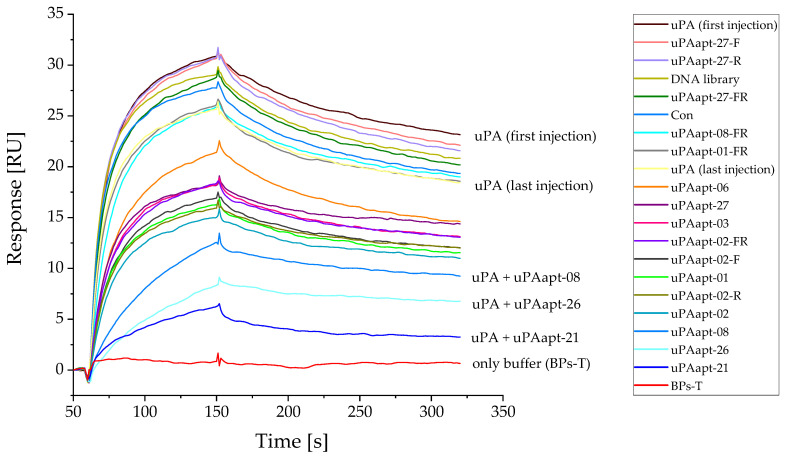
Screening of aptamers inhibiting uPA−uPAR binding. SPR sensograms showing each response unit (RU) over the time (s) for 20 nM human uPA alone or together with individual aptamers (500 nM) injected from 60 to 150 s on a sensor surface with immobilized human uPAR. As the ability of uPA binding to uPAR decreases due to the regeneration between each sample injection, binding of uPA alone was checked before and after aptamer injections.

All full-length aptamers as well as selected, truncated variants were screened for their ability to inhibit uPA-uPAR binding, where several aptamers were able to inhibit binding of uPA to uPAR. Although they differed in the strength of inhibition, the following aptamers showed an inhibitory effect, beginning with the aptamer that showed the best inhibitory effect: uPAapt-21, uPAapt-26, uPAapt-08, uPAapt-02, uPAapt-01, uPAapt-02-R, uPAapt-02-F, uPAapt-02-FR, uPAapt-03, uPAapt-27 and uPAapt-06. Besides some aptamers that showed no inhibitory effect (uPAapt-01-FR, uPAapt-08-FR, uPAapt-27-FR, uPAapt-27-R and uPAapt-27-F), the DNA-library used for aptamer selection as well as the sequence-unrelated control aptamer Con also showed no inhibitory effect. For the SPR sensogram of the screening of aptamers inhibiting uPA-uPAR binding, see Figure A1. The aptamers that showed the best inhibitory effect (uPAapt-21, uPAapt-26 and uPAapt-08) were used for IC_50_ calculation. The control aptamer Con was also included in IC_50_ calculation to verify sequence-specific IC_50_ values. Binding of uPA to immobilized uPAR on the sensor surface in the presence of increasing concentrations of the different aptamers is shown in Figure 5.

**Figure 6:**
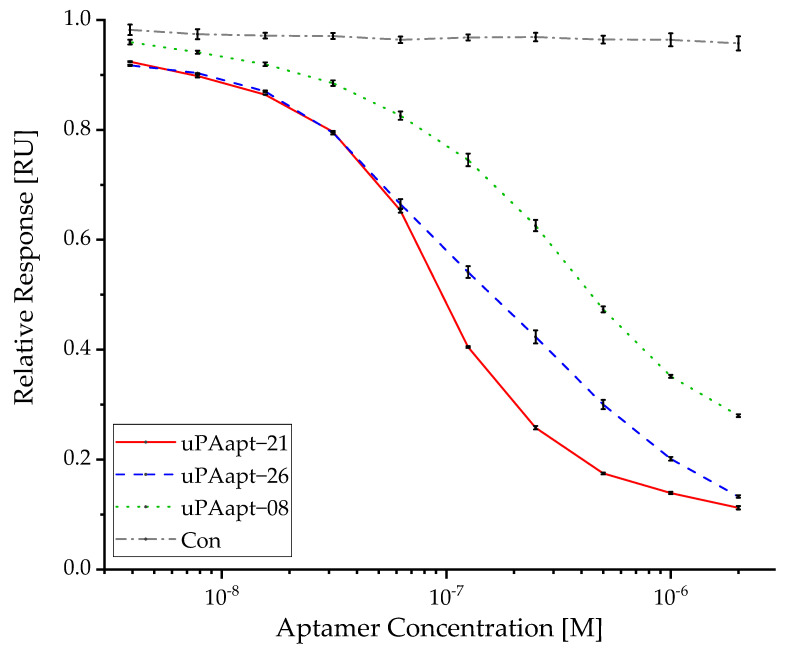
Reported uPA capture level after injection of each sample was plotted as a function of the aptamer concentration (3.9–2000 nM) relative to the response of 20 nM uPA alone for uPAapt−21, uPAapt−26, uPAapt−08 and the sequence-unrelated control aptamer Con. For the calculated IC_50_ values, see Table 3. The relative response (RU) for each sample is shown as the mean value of independent experiments. Error bars represent the range of measured values. Number of records *n* = 2.

While uPAapt-21, uPAapt-26 and uPAapt-08 showed a dose-dependent effect on the inhibition of uPA binding to uPAR on the sensor surface, the sequence-unrelated control aptamer Con showed no inhibitory effect. A slight decrease in the reported response values for Con can be attributed to repeated regenerations after each sample injection, which leads to a decrease in uPA-uPAR binding. No binding of aptamers (125–2000 nM) to uPAR could be detected. Response units of uPA in complex with different concentrations of each aptamer were plotted as a function of aptamer concentrations relative to the response of uPA without an aptamer (Figure 6).

As repeated regeneration steps were conducted, binding of uPA to the sensor surface immobilized with uPAR reduced over the measurements. To include this in IC_50_ calculation, uPA was injected before each concentration series of the different aptamers and the loss of the signal was fitted using a polynomial regression model. Each uPA-aptamer-complex response was reduced by the calculated loss of signal. Based on these data, IC_50_ values were estimated by a nonlinear regression analysis, fitting the data to a dose-response function. IC_50_ values with their standard error for the inhibition of uPA-uPAR binding by the different aptamers are shown in Table 3.

### 2.5. Proteolytic Activity of uPA Can Be Inhibited by Several Aptamers

**Figure 7:**
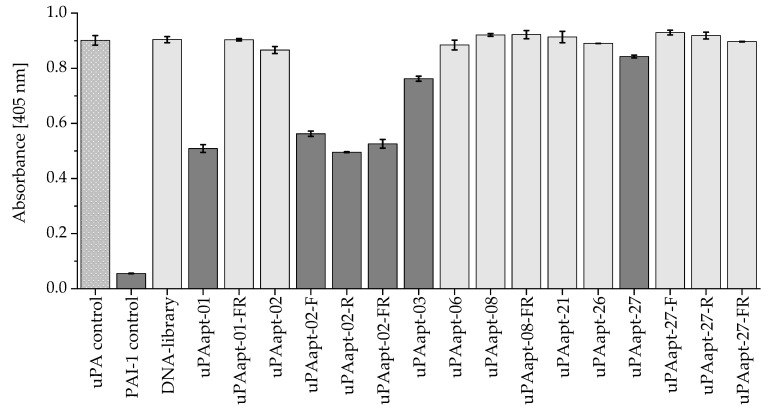
uPA inhibition assay for various aptamers. uPA activity was detected by hydrolysis of the chromogenic substrate. The absorbance was measured at 405 nm after 4 h at 37 °C for uPA (120 pmol) alone (uPA control) or incubated with various aptamers (600 pmol). Incubation with PAI−1 was carried out as a positive control for the specific inhibition of uPA. The DNA-library was used as a control to demonstrate sequence-dependent inhibition. The PAI−1 control and aptamers that showed inhibition of the uPA activity are highlighted in dark grey. The absorbance, for each sample, is shown as the mean value of technical replicates. Error bars represent the range of measured values. Number of records *n* = 2.

All full-length aptamers and various truncated variants were screened for their ability to inhibit the proteolytic activity of uPA using the uPA inhibition assay. Although the various aptamers showed only partial inhibition of the proteolytic activity of uPA, uPAapt-01, uPAapt-02-F, uPAapt-02-R, uPAapt-02-FR and uPAapt-03 showed inhibition of uPA activity compared to uPA alone. Moreover, uPAapt-27 showed a very small effect on uPA activity. In particular, uPAapt-01, uPAapt-02-F, uPAapt-02-R and uPAapt-02-FR could inhibit the proteolytic activity of uPA by at least 35% (uPAapt-01: 44%, uPAapt-02-F: 38%, uPAapt-02-R: 45%, uPAapt-02-FR: 42%, uPAapt-03: 15%, uPAapt-27: 6%). For the calculation, uPA without an aptamer was taken as 0% inhibition. All other tested aptamers and several truncated variants, as well as the ssDNA library used as a control, showed no effect on uPA activity compared to uPA alone (Figure 7).

**Figure 8:**
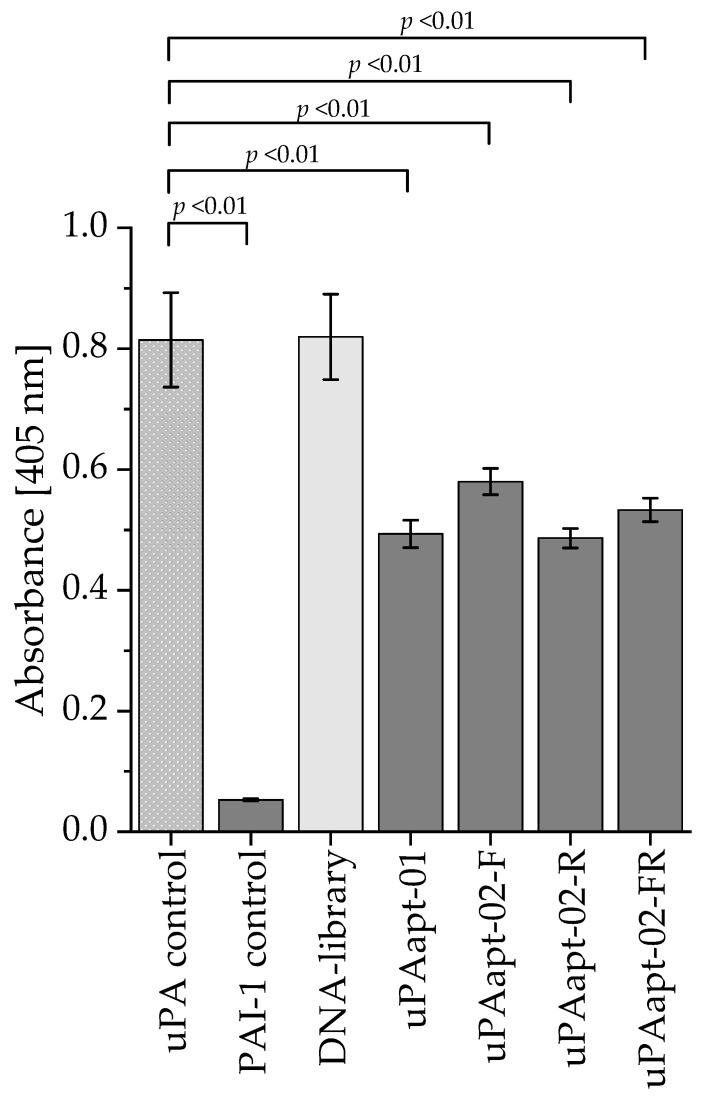
uPA inhibition assay for uPAapt−01 and truncated versions of uPAapt−02 (uPAapt−02−F, uPAapt−02−R and uPAapt−02−FR). uPA activity was detected by hydrolysis of the chromogenic substrate. The absorbance was measured at 405 nm after 4 h at 37 °C for uPA (120 pmol) alone (uPA control) or incubated with aptamers (600 pmol). Incubation with PAI−1 was carried out as a positive control for specific inhibition of uPA. The DNA-library was used as a control to demonstrate sequence-dependent inhibition. PAI−1 control and tested aptamers showed significant inhibition of the uPA activity (*p* <0.01). The absorbance for each sample is shown as the mean value of three independent experiments (number of records *n* = 3). Error bars represent the standard deviation. *p*-values were determined by Welch’s *t*-test.

To verify whether there was statistically significant inhibition of uPA activity by uPAapt-01 and the truncated variants of uPAapt-02, two additional independent experiments were performed for uPAapt-01, uPAapt-02-F, uPAapt-02-R and uPAapt-02-FR to achieve a total of three independent experiments. Again, uPA alone, uPA incubated with PAI-1 or the DNA-library were included as controls. Statistically significant inhibition (*p* < 0.01) of uPA activity could be demonstrated for uPAapt-01, uPAapt-02-F, uPAapt-02-R and uPAapt-02-FR compared to uPA alone (Figure 8). Significance was determined by Welch’s *t*-test.

**Figure 9:**
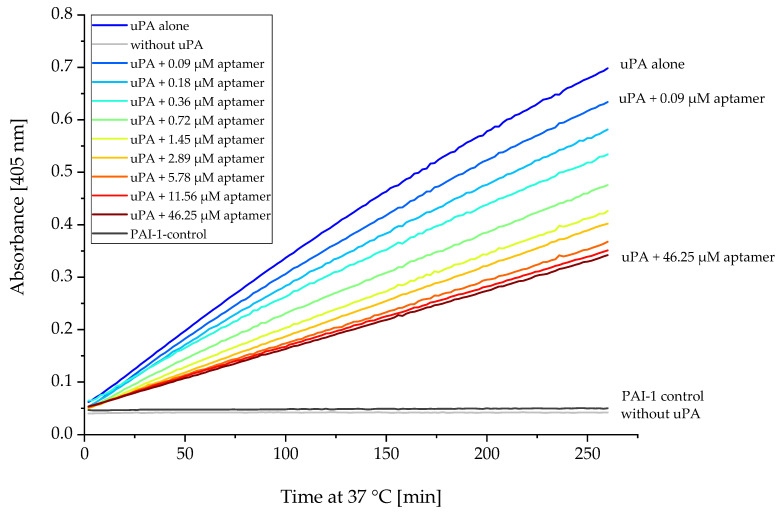
uPA activity inhibition by uPAapt−02−FR. uPA activity was measured by the hydrolysis of the chromogenic substrate. Absorbance was measured at 405 nm over 2 h 20 min at 37 °C in the presence of increasing concentrations of uPAapt−02−FR (0.09, 0.18, 0.36, 0.72, 1.45, 2.89, 5.78, 11.56 and 46.25 µM). Here, uPAapt−02−FR showed a dose-dependent effect on the inhibition of uPA activity, with a substantial reduction in the conversion of the chromogenic peptide substrate. While samples added with PAI−1 served as a positive control, uPA (0.46 µM) alone was used as a negative control. Samples that did not contain uPA were also included as controls. The absorbance for each sample is shown as the mean value of technical replicates. Number of records *n* = 2.

As several aptamers showed similar inhibitory effects, uPAapt-02-FR was selected for further characterization. As a representative aptamer, uPAapt02-FR showed a dose-dependent effect on the inhibition of uPA activity with a substantial reduction in the conversion of the chromogenic peptide substrate in the range of 0.09 to 46.25 µM. Here, the highest concentration of uPAapt-02-FR (46.25 µM) corresponded to a 100-fold excess of aptamer (Figure 9).

## 3. Discussion

In this study, we present novel ssDNA aptamers that can not only target different uPA forms and block the interaction of uPA with its receptor but also inhibit the proteolytic activity of uPA. To the best of our knowledge, no other aptamers that can bind in the region of the SPD and inhibit uPA activity have been reported to date. Dupont et al. [19] demonstrated that a 2′-F-pyrimidine-modified RNA aptamer with high affinity and specificity can inhibit the accumulation of uPA-catalyzed plasminogen activation on cell surfaces by binding to the GFD of uPA and thereby blocking uPA-uPAR interaction. None of their selected aptamers were reported to bind near to or at the serine protease domain and thereby block uPA activity [19]. Through binding of the 2′-fluoro-pyrimidine-modified RNA aptamers upanap-126 and upanap-231 from Botkjaer et al. [20] to the catalytic domain of pro-uPA, the proteolytic conversion of pro-uPA to the active two-chain HMW-uPA form was inhibited, but no inhibition of plasminogen activation could be achieved. This suggests that binding inhibited pro-uPA activation rather than direct uPA-mediated plasminogen activation. However, upanap-126 significantly affected tumor cell intravasation and dissemination in vivo due to the inhibition of pro-uPA activation, competing with the binding of pro-uPA to uPAR and preventing the binding of the pro-uPAR/uPAR complex to the glycoprotein vitronectin [20].

We selected novel ssDNA aptamers against HMW- and LMW-uPA that—in addition to binding to the ATF of uPA, which is already known from other aptamers—also bind in the region of the catalytic domain. Several different aptamers were identified that showed binding either to HMW-uPA or to both uPA forms using different characterization methods [23]. Hence, we assumed that aptamers must have different binding sites. However, results for some aptamers vary based on the method used to determine binding characteristics, and therefore, are not explicit for every aptamer. EMSA is a label- and immobilization-free method to detect binding to protein targets in a cost-effective, easy and quick way. However, this qualitative method tends to detect only binders with high affinity to their target. According to the manufacturer, the fluorescent dye Invitrogen™ Quant-iT™ OLIGREEN™ (Thermo Fisher Scientific Inc., Waltham, MA, USA), which was used in the FLAAs, exhibits significant base selectivity. Therefore, intercalation of the fluorescent dye is primarily sequence-dependent. Additionally, the structure of aptamers will probably also influence the intercalation of the dye. This must be accounted for when evaluating these methods since the sequence of an aptamer and its associated structure play a very important role in binding to the target. Here, it may be that some aptamers stain particularly well, while others do not, which seems to be independent of binding strength to the target. As a result, well-binding sequences may be lost in FLAAs.

Therefore, other quantitative methods like SPR or MST, which are more sensitive, were used. However, quantitative methods also have limitations. In SPR experiments, aptamers are immobilized preferably on the sensor surface, which can affect the access to the aptamer binding site and the aptamer structure. Both can be influenced, for example, by the coupling position of the aptamer (5’- or 3’-end or any internal position), additionally used spacer molecules or non-specific interactions with the matrix surface. While uPAapt-03 and uPAapt-27 showed binding to LMW-uPA in FLAA experiments, no binding was detected in SPR. Loss of binding may indicate that immobilization of the aptamer impacts the binding activity of uPAapt-03 and uPAapt-27 to LMW-uPA. The proposal that aptamers can bind LMW-uPA by various methods may be confirmed by their ability to inhibit uPA activity. In particular, uPAapt-01, uPAapt-02 and uPAapt-03 showed binding to LMW-uPA in the region of the serine protease domain, which was confirmed by the inhibitory effect on the uPA activity. In addition, uPAapt-27 showed a very small effect on the catalytic activity, which confirms that it likely binds near the catalytic domain and affects enzymatic cleavage through steric hindrance. In addition to classical competitive inhibition, binding between the aptamer and target protein can affect protein folding itself, resulting in a type of non-competitive inhibition. Interestingly, uPAapt-01 showed binding to LMW-uPA, while its truncated version uPAapt-01-FR only bound to HMW-uPA and no longer inhibited uPA activity. This is a good example of how the constant primer regions of aptamers may affect the structure and thus also the binding properties. Here, the part that also binds to LMW-uPA might have been cut off. The structure of an aptamer is in a chemical equilibrium of the most energetically favorable conformation. Therefore, an aptamer can form different structures whereby one structure is usually predominant in a buffer solution. This could be another explanation, i.e., that the LMW-uPA binding structure can likely no longer be formed since different structures are formed within one sequence. Accordingly, uPAapt-03 and uPAapt-27 showed no further binding to LMW-uPA in SPR since the sequences have a certain orientation due to immobilization on the sensor surface, and thus, also a certain structure.

Although uPAapt-21 also showed binding to both uPA-forms by various methods, it could not inhibit uPA activity. Interestingly, uPAapt-21 showed the best inhibitory effect on uPA-uPAR binding. While we were unable to determine a specific binding site, we hypothesize that binding of uPAapt-21 results in a steric hindrance near the GFD on HMW-uPA, which is responsible for binding to uPAR. Previously, the size and shape of an aptamer and the domain organization of a multi-domain protein like uPA were shown to provide the basis for extensive sterical interference with protein-ligand interactions [24,25]. Dupont et al. showed that the effect of an aptamer does not always depend on the binding site, which can be somewhere else [24,25]. Here, epitope mapping of the different aptamers by microarray or X-ray crystallography and NMR-spectroscopy could provide information on different binding epitopes or different structural formations of the aptamer-uPA complex [26]. Aptamers such as uPAapt-08 and uPAapt-26, which showed distinct binding only to HMW-uPA and were, therefore, assumed to bind to ATF, showed an inhibitory effect on uPA-uPAR binding.

Overall, we could select novel ssDNA aptamers with different binding behaviors and inhibitory effects either attributable to the binding regions or the structure that binds to uPA. Inhibition of uPA activity and the mere binding of uPA to its receptor uPAR is an important task to prevent tumor growth, invasion and metastasis [4,5]. While some aptamers showed no inhibitory effect or only inhibited uPA-uPAR binding, all aptamers inhibiting uPA activity showed additional inhibition of the formation of the uPA-uPAR complex, even if not as strong as those that can only inhibit uPA-uPAR binding. A uPA-directed inhibitor that blocks formation of the uPA-uPAR complex and uPA activity is of great advantage as it has been shown that most blocking agents serve as antagonists to uPA-uPAR interaction by binding to uPAR, which still induces the downstream interactions of the uPA-uPAR complex [19]. Using synthetic or naturally occurring inhibitors like aptamers with low molecular weight is also a more cost-effective and feasible approach than the use of antibodies, and expression of PAI-1 is hampered by poor pharmacokinetics and pharmacodynamics [8]. Since our selected aptamers showed different properties affecting uPA, they may serve as promising agents in therapeutics as they interfere with the pathological function of the uPA system.

However, our studies were only performed in situ and inhibition of uPA activity was tested only with an artificial chromogenic substrate. Looking ahead, it would be interesting to determine whether the aptamers can also prevent plasminogen activation in solution, as well as cell surface-associated plasminogen activation, and whether they inhibit binding of uPA to uPAR on cell surfaces to inhibit associated downstream processes. To see if the use of these aptamers inhibits tumor cell invasion in vitro and in vivo would also be of great interest. In addition, many new questions arise: whether the aptamers also affect uPA-PAI-1 binding or plasmin-catalyzed pro-uPA activation, or if aptamers can block receptor-mediated endocytosis of the uPA-PAI-1 complex. However, since DNA is susceptible to nucleases, before in vitro or in vivo use, it may be expedient to stabilize the aptamers against nuclease degradation, for example, by chemical modification [27]. In addition to their use in therapy, they could also be of interest for analytics due to their simple synthesis and modifications, as uPA is also used as a prognostic and diagnostic marker. Since aptamers showed different binding sites, their use in different assay formats—especially in a sandwich assay format for detecting uPA—would be of particular interest.

## 4. Materials and Methods

### 4.1. Target Preparation

**Figure A2:**
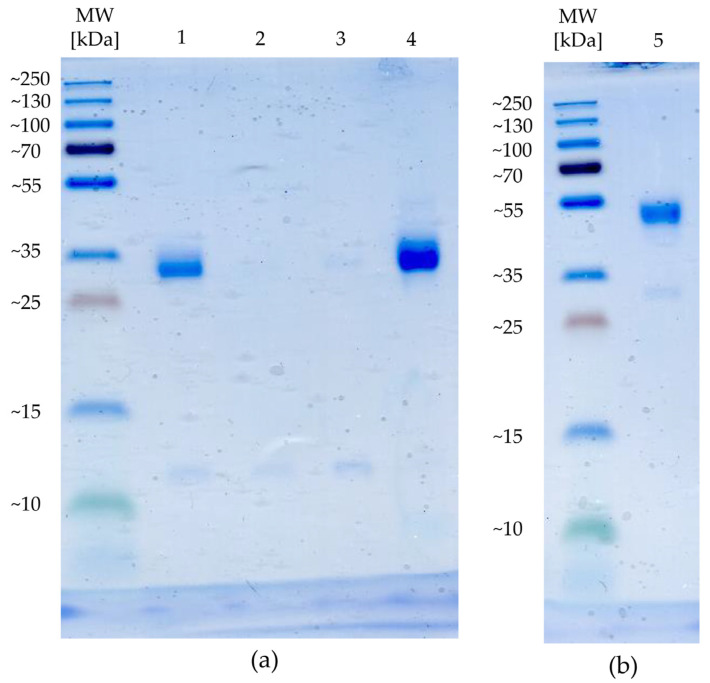
Analysis of the uPA forms by SDS-Page, whereby 1.5 µg of the different uPA forms was applied for analysis. (**a**) Complete conversion to LMW−uPA after incubation (1), first and second flow-through of the Nanosep^®^ Centrifugal Device with Omega™ Membrane 30 K (2,3) and purified and concentrated LMW−uPA (4). (**b**) Analysis of HMW−uPA (5).

Native high-molecular-weight uPA (HMW-uPA) isolated from human urine was purchased from ProSpec-Tany TechnoGene Ltd. (Ness-Ziona, Israel). To select aptamers against both uPA forms, the low-molecular-weight form of uPA (LMW-uPA) was obtained by autocatalytic conversion [28]. For this purpose, HMW-uPA was dissolved in PBS at a concentration of 1.0 µg/µL and incubated at 37 °C for a minimum of 32 days. To obtain 33 kDa LMW-uPA, the sample was purified and concentrated using a Nanosep^®^ Centrifugal Device with Omega™ Membrane 30 K (Pall Corporation, New York, NY, USA). Complete conversion and purity of LMW-uPA were determined by SDS-gel electrophoresis [29] (Figure A2). For further use, both uPA forms were biotinylated separately using EZ-Link™ Sulfo-NHS-SS-Biotin (Thermo Fisher Scientific Inc., Waltham, MA, USA) according to the manufacturer’s instructions.

### 4.2. Oligonucleotides and Semiautomatic In Vitro Selection

A single-stranded DNA oligonucleotide library with a theoretical diversity of 1.9 × 10^25^ was used as a precursor pool for aptamer selection. A 42-nt random sequence was flanked by a constant 20-nt primer region on each side (5′-AGGTAGAGGAGCAAGCCATC-(N42)-GATGCGTGATCGAACCTACC-3′). Aptamers were selected using a semiautomatic process as previously described [30]. Before selection, ssDNA was refolded. To do so, the ssDNA-library was heated to 90 °C for 3 min and then slowly (for about 30 min) cooled to 25 °C in binding buffer BPs-T (50 mM Bis-Tris/HCl pH 6.5, 110 mM NaCl, 5 mM MgCl_2_, 1 mM CaCl_2_, 1 mM KCl, 0.05% *v/v* Tween^®^ 20, Carl Roth GmbH + Co. KG, Karlsruhe, Germany). The ssDNA library was obtained from three different providers (Noxxon Pharma AG, Berlin, Metabion GmbH, Planegg, AptaIT GmbH; Planegg, Germany). A molar mixture from these three different providers (25% from Noxxon Pharma AG, 25% from Metabion GmbH and 50% from AptaIT GmbH) was used for the first round of selection to work with an optimized equal base distribution on every randomized base position. To select aptamers against HMW- and LMW-uPA, selection was carried out separately for each uPA form. To do so, the refolded ssDNA was incubated separately with the biotinylated uPA forms for 60 min at room temperature in BPs-T. After incubation, biotinylated LMW- or HMW-uPA with the bound ssDNA was coupled with streptavidin-coated magnetic beads (Dynabeads M-280 streptavidin, Invitrogen Dynal AS, Oslo, Norway) to transfer them automatically for several washing steps. From selection round six, remaining streptavidin moieties were blocked with 20 µM biotin in BPs-T. To simplify the selection of matrix binders, preselection steps with uncoupled streptavidin beads (from selection round two) and biotin-blocked streptavidin beads (from selection round six) were carried out before uPA incubation. Extensive washing steps with the same buffer were performed after coupling to streptavidin-coated beads (and after blocking with biotin) to remove non-binding oligonucleotides. To observe specific enrichment of uPA-binding DNA sequences, uncoupled streptavidin beads were included in certain rounds of selection as a control. The uncoupled beads were treated separately under the same SELEX conditions. DNA sequences binding to the beads were eluted non-specifically with 8 M urea in BPs-T for 30 min at 65 °C and 900 rpm using a thermoshaker. From selection round six, an additional specific elution step was introduced—with 500 mM dithiothreitol (DTT) in BPs-T for 60 min—before unspecific elution, to reduce the disulfide bond of the EZ-Link™ Sulfo-NHS-SS-Biotin linked to the uPA-forms. Eluted fractions were precipitated with 2-propanol at −20 °C overnight. Precipitated DNA was amplified by PCR with 5′-AGG TAG AGG AGC AAG CCA TC-3′ used as the forward primer and 5′-biotin-C6-GGT AGG TTC GAT CAC GCA TC-3′ used as the reverse primer, which allowed for the subsequent purification of the aptamer strand by alkali denaturation on streptavidin magnetic beads [30]. Finally, after up to 12 selection rounds with the two uPA forms, enriched DNA pools of different selection rounds were sequenced using the next-generation sequencing (NGS) technique by GATC Biotech (Ebersbach, Germany), and the following sequence analysis was performed with COMPAS analysis software (AptaIT GmbH, Planegg, Germany).

### 4.3. Electrophoretic Mobility Shift Assay (EMSA)

To detect binding between the aptamer and target protein, an electrophoretic mobility shift assay [31] was carried out under native conditions on a 12% polyacrylamide gel (native PAGE) containing 0.5 x Tris-borate buffer (without EDTA) and 1 mM MgCl_2_. Aptamers were refolded in BPs-T as described above. For interaction analysis of each uPA-form and aptamer, 45 pmol of each uPA form was incubated with 15 pmol of aptamer (for a 3:1 ratio) in a total volume of 15 µL BPs-T for 60 min at 300 rpm and 23 °C on a thermoshaker. Each aptamer (15 pmol), alone without protein, in a total volume of 15 µL BPs-T was carried along as a control. The aptamer alone and the aptamer-protein mixture were subjected to electrophoresis. To detect the difference between the free nucleic acid that migrates faster than the corresponding mixture if a protein-nucleic acid complex is formed, the polyacrylamide gel was stained with ethidium bromide and samples were visualized under UV light.

### 4.4. Fluorescent Dye-Linked Aptamer Assay (FLAA)

Fluorescence-based binding assays were carried out on a black Pierce™ streptavidin-coated high-capacity plate (Thermo Fisher Scientific Inc., Waltham, MA, USA) as previously described [32]. Before immobilization and between incubation steps, wells were washed three times with 300 µL BPs-T for 90 s at 300 rpm in a thermoshaker. The different biotinylated uPA forms (35 pmol) were immobilized in 100 µL BPs-T for 60 min in different wells. Subsequently, wells were incubated with 50 µM biotin in 200 µL BPs-T for 30 min to block remaining biotin-binding residues and provide the same surface structure as in the selection procedure on magnetic beads. Biotin-blocked wells without uPA were used as a no-target control (NTC). Before application, aptamers were refolded as previously described, and 32 pmol of refolded ssDNA in a total volume of 100 µL BPs-T was added to each well and incubated for 60 min at 23 °C. After washing for 90 s at 300 rpm, 100 µL of a 1:200 dilution of Invitrogen™ Quant-iT™ OLIGREEN™ (Fisher Scientific GmbH, Schwerte, Germany) in BPs-T was added to each well. Following an incubation step of 12 min, fluorescence (excitation 485 nm, emission 535 nm) was measured by multimode microplate reader Mithras^2^ LB 943 Monochromator Multimode Reader (Berthold Technologies GmbH & Co. KG, Bad Wildbad, Germany). Due to space limitations, binding of the aptamers was tested using different microtiter plates. Therefore, signals of the DNA-library used as a control in each experiment were averaged from all experiments, and all aptamers were presented in one diagram. All aptamers were tested as duplicates.

### 4.5. Surface Plasmon Resonance Spectroscopy

Surface plasmon resonance (SPR) spectroscopy experiments [23] were performed using a Biacore™ T200 (GE Healthcare Bio-Sciences AB, Uppsala, Sweden)) at an operating temperature of 25 °C. To analyze the binding characteristics of aptamers to uPA forms and determine dissociation constants (K_D_s), biotinylated aptamers were immobilized as ligands on the sensor chip surface, while uPA forms served as analytes. Neutravidin was immobilized on an EDC/NHS-activated HC200M SPR Sensor Chip (XanTec bioanalytics GmbH, Düsseldorf, Germany) using a Biacore™ immobilization wizard at a flow rate of 20 µL/min. For this purpose, 0.2 M EDC and 0.1 M NHS were dissolved in 50 mM MES (2-(N-morpholino) ethane sulfonic acid) pH 5 and injected for 3 min. After activation, 20 µg/mL neutravidin dissolved in 10 mM acetate buffer pH 5.5 was injected for 2 min, followed by washing with double-distilled water (ddH_2_O) for 10 min. Subsequently, remaining binding sites were blocked with 1 M ethanolamine for 7 min. To determine association and dissociation rates, as well as K_D_s of aptamer-uPA complexes, 5′-biotinylated aptamers were immobilized on neutravidin-coated HC200M sensor surfaces to a level of approximately 100 RU. For this, biotinylated aptamers (100 nM) were prepared in running buffer (BPs-T containing 0.005% *v/v* Tween^®^ 20 instead of 0.05% Tween^®^ 20, Carl Roth GmbH + Co. KG, Karlsruhe, Germany) and refolded as previously described. Each flow channel was immobilized with a different biotinylated aptamer, respectively, while channel one served as a reference channel without an aptamer. A sequence-unrelated control oligonucleotide (Con) with the sequence 5′-GGG AAT TCG AGC TCG GTA CCG TAC AGT ACT GCA TAT CTC ATA CTT CCT AGA TAC CAT CCC TGC AGG CAT GCA AGC TTG G-3′ was additionally analyzed for comparison and to demonstrate sequence-dependent binding. Remaining biotin-binding sites on charged channels and the reference channel were blocked with excess biotin (40 µM) for 2 min. Both uPA-forms, HMW- and LMW-uPA, were diluted in running buffer and concentrations ranging from 3.9 to 1 µM were applied on each flow channel at a flow rate of 30 µL/min with 3 min injections and a 5-min delay. The chip surface was regenerated by applying 20 mM Na_2_CO_3_ for 30 s at a flow rate of 10 µL/min.

The uPA-uPAR inhibitory activity of the aptamers in SPR experiments was determined as previously described [19]. Here, human uPAR (human uPAR/CD87 protein, Sino Biological Inc., Beijing, China) was immobilized on an EDC/NHS-activated HC200M SPR Sensor Chip (XanTec bioanalytics GmbH, Düsseldorf, Germany) to approximately 500 RU using a Biacore™ immobilization wizard at a flow rate of 10 µL/min. The reference surface and remaining binding sites were blocked with 1 M ethanolamine for 7 min. Human uPA (HMW-uPA) alone (20 nM) or together with refolded aptamers in different concentrations (3.9–2000 nM) was prepared in BPs-T and passed over the sensor surfaces at a flow rate of 30 µL/min for 90 s and a 3-min delay. Aptamers alone (125–2000 nM) were injected as well, to exclude the binding of aptamers to uPAR. The chip surface was regenerated with 100 mM acetic acid (pH 2.8) containing 0.5 M NaCl for 15 s at a flow rate of 20 µL/min. Before determination of IC_50_ values, all aptamers were screened for uPA-uPAR inhibitory activity by injecting uPA alone (20 nM) or together with individual aptamers (500 nM) over the sensor surface. All interactions were analyzed using Biacore™ BIAevaluation software (version 3.2, GE Healthcare Bio-Sciences AB, Uppsala, Sweden). Aptamers that showed the best results for inhibition of uPA binding to uPAR were selected for IC_50_ calculation. Response values of uPA in complex with different concentrations of each aptamer were plotted as a function of aptamer concentrations relative to the response of uPA without an aptamer. As repeated regeneration steps were conducted, binding of uPA to the sensor surface immobilized with uPAR reduced over the measurements. To include this in IC_50_ calculation, uPA was injected before each concentration series of the different aptamers, and loss of the signal was fitted using a polynomial regression model. Each uPA-aptamer-complex response was reduced by the calculated loss of signal. IC_50_ values were estimated by a nonlinear regression analysis, fitting the data to a dose-response function using OriginPro 2021 software (OriginLab Corporation, Northampton, MA, USA). The sequence-unrelated control oligonucleotide Con was analyzed for comparison and to demonstrate sequence-dependent inhibition. Two independent experiments were performed. The mean values of the relative response with the respective standard deviation were used for IC_50_ value determination.

### 4.6. Microscale Thermophoresis

Microscale thermophoresis (MST) experiments were performed on a Monolith NT.115 (NanoTemper Technologies, Munich, Germany) using standard capillaries. Measurements were performed at an MST power of 60% and excitation power ranging from 20 to 60%. In our work, 5′-cyanine 5-labeled aptamers were diluted to 20 nM in BPsT, refolded as previously described and used as the fluorescently labeled binding partner at a constant concentration. Serial dilutions of HMW- and LMW-uPA ranging from 0.1 to 333 nM were prepared in BPs-T. For pro-uPA (BioVision Inc., Milpitas, USA), mouse uPA (Abcam plc, England) and tissue-type plasminogen activator (tPA, EMD Millipore Corp., Burlington, VT, USA) concentrations ranged from 0.1 to 500 nM. In the research, 10 µL of refolded aptamer and protein dilutions was mixed and incubated for 30 min. Samples were measured by the Monolith NT.115. The recorded fluorescence was normalized to the Fnorm per mill and fitted utilizing the K_D_ formula derived from the law of mass action by MO Affinity Analysis software (NanoTemper Technologies GmbH, Munich, Germany) version 2.3 [33,34]. All measurements were performed as triplicates.

### 4.7. uPA Inhibition Assay

Inhibition of uPA activity was tested using a Chemicon^®^ PAI Activity Assay Kit (Merck KGaA, Germany) according to the manufacturer’s specifications with some modifications. Here, 10x BPs-T (500 nM Bis-Tris/HCl pH 6.5, 1.1 M NaCl, 50 mM MgCl_2_, 10 mM CaCl_2_, 10 mM KCl, 0.5% *v/v* Tween^®^ 20, Carl Roth GmbH + Co. KG, Karlsruhe, Germany) was used as the assay buffer (15 µL for the aptamer samples and 20 µL for control samples). Individual aptamers were tested in a five-fold excess for inhibition of enzyme activity to obtain a ratio (aptamer:target) of 5:1. For each well, 600 pmol of aptamer was refolded in 50 µL BPs-T. Then, 10 µL 0.65 µg/µL uPA from the assay kit (equivalent to 120 pmol), alone or in the presence of individual refolded aptamers, was preincubated for 30 min at 23 °C and 350 rpm in a microtiter plate. DdH_2_O was added to each sample to reach a total volume of 180 µL before preincubation. For the control (uPA alone), the aptamer solution was replaced by 50 µL ddH_2_O. For the uPA negative control, uPA was replaced by 10 µL ddH_2_O. Activated plasminogen activator inhibitor (PAI-1) was prepared as described in the manufacturer’s protocol and was used as a positive control. Here, the aptamer solution was replaced by 40 µL activated PAI-1 solution and 10 µL ddH_2_O. To test inhibition by uPAapt-02-FR at various concentrations over time, a serial dilution of refolded uPAapt-02-FR ranging from 0.36 to 185 µM was prepared in BPs-T, and 50 µL of each dilution (equivalent to 18–9250 pmol) was preincubated with 10 µL uPA (0.5 µg/µL) from the assay kit (equivalent to 92.5 pmol) as previously described. Afterward, 20 µL of the chromogenic substrate (2.5 µg/µL tripeptide with a p-nitroanilide (pNA) group) was added to each well and hydrolysis was monitored by measuring the absorbance at 405 nm using the EnVision^®^ 2105 multimode plate reader (PerkinElmer^®^ Inc., Waltham, MA, USA). Samples were measured after incubation at 37 °C for 4 h or every 2 min at 37 °C over 4 h 20 min. Significant inhibition of the uPA activity by the different aptamers compared to uPA alone was tested by Welch’s unequal variances t-test. Samples were tested as duplicates.

## 5. Conclusions

In this study, we demonstrated the selection of novel, highly affine and specific DNA aptamers up to 82-nt long by a semi-automatic in vitro selection process (SELEX), which can bind to different forms of human urokinase. Since they bind to different uPA forms, we can differentiate between high-molecular-weight uPA (HMW-uPA) and low-molecular-weight uPA (LMW-uPA). Therefore, we assume that our aptamers bind to different regions of uPA. High affinity and specificity for human uPA, as well as for the zymogen pro-uPA, was demonstrated by surface plasmon resonance (SPR) and microscale thermophoresis (MST). Furthermore, we found that aptamer binding inhibited the binding of uPA to its receptor uPAR and the proteolytic activity of uPA. Since uPA is a much-discussed marker for prognosis and diagnosis in various types of cancer, as well as a target for cancer therapies, our aptamers represent a promising tool for diagnostic applications and could serve as promising agents in therapeutics.

## 6. Patents

All aptamers used in this manuscript are protected by pending patents.

## Figures and Tables

**Table 1:** Sequences of the variable region of the aptamers binding uPA. Each 41−nt and 42−nt long variable region is flanked by the constant regions of the 20−nt long forward− (5′−AGGTAGAGG-AGCAAGCCATC−3′) and reverse− (5′−GATGCGTGATCGAACCTACC−3′) primer binding sites.

Name	Variable Region (5′−3′)
uPAapt−01	ATGGTAACATGCACTAGGTCGCGATGGTTCCGTCTGATCGCT
uPAapt−02	CAAGCGGGGGTGAGAGATCTGTCAGTACGAGCTGGGTTTGCG
uPAapt−03	ACGGAGGCAGAGGGTGAGAGATCTGTTAGTACTAGCCCATGT
uPAapt−06	GGGTGGGTGGGTGGGTGATGGCTCGACTCTACCCTGGCGCTG
uPAapt−08	CAGCGGTAGGGGTTATATAGCTGCGCCATAGGGTACTCGTG
uPAapt−21	GGAGGTACTCACCGACGCTGAACTCCATAGAATGTGGTGATG
uPAapt−26	ATCCGTGGCGTGGTTGGTGGGGGAGGTGGGCGGGTAGACGGT
uPAapt−27	ATGGGGTGTTATGGGGGGGGCTTATTGCGGTCAGGGGACGAT

**Table 2:** Dissociation constants (K_D_) including the K_D_ confidence from MST measurements for uPAapt−02−FR and uPAapt−21 with HMW−uPA and LMW−uPA, and additionally, for uPAapt−02−FR with pro−uPA, mouse−uPA and tPA.

Aptamer	Target	K_D_ (M)	K_D_ Confidence (M)
5′−Cy5−uPAapt−02−FR	HMW−uPA	6.69 × 10^−9^	±1.55 × 10^−9^
	LMW−uPA	9.40 × 10^−9^	±3.10 × 10^−9^
	pro−uPA	1.40 × 10^−8^	±0.65 × 10^−8^
	mouse uPA	-*	-*
	tPA	-*	-*
5′−Cy5−uPAapt−21	HMW−uPA	2.08 × 10^−7^	±0.63 × 10^−7^
	LMW−uPA	1.41 × 10^−6^	±0.45 × 10^−6^

Note: -*, no binding detected.

**Table 3:** IC_50_ values of selected aptamers for inhibition of interaction between uPA and its receptor uPAR. IC_50_ values were estimated by nonlinear regression analysis, fitting the data to a dose-response function.

Aptamer	IC_50_ (±Standard Error) (M)
uPAapt−21	8.70 × 10^−8^ (±0.40 × 10^−8^)
uPAapt−26	1.65 × 10^−7^ (±0.16 × 10^−7^)
uPAapt−08	2.98 × 10^−7^ (±0.17 × 10^−7^)
Con	-*

Note: -*, no inhibition could be detected.

## Data Availability

The datasets for the current study are available from the corresponding author on request.

## Appendix A

**Table A1:** Results of electrophoretic mobility shift assay (EMSA) for HMW−uPA and LMW−uPA.

Aptamer	EMSA	
	HMW−uPA	LMW−uPA
uPAapt−01	+	+
uPAapt−02	+	+
uPAapt−03	+	(+)
uPAapt−06	+	-
uPAapt-08	+	-
uPAapt−21	+	-
uPAapt−26	+	-
uPAapt−27	+	-

Note: + = binding, - = no binding, (+) = only light shift detected.

**Table A2:** On- and off-rates (k_a_ and k_d_) including chi^2^ and dissociation constants (K_D_s) from SPR measurements.

Aptamer	Target	k_a_ [M^−1^ s^−1^]	K_d_ [s^−1^]	K_D_ [M]	Chi^2^
uPAapt−01	HMW−uPA	6.83 × 10^5^	5.15 × 10^−4^	7.54 × 10^−10^	1.48
	LMW−uPA	7.24 × 10^4^	1.57 × 10^−3^	2.17 × 10^−8^	0.02
uPAapt−01−FR	HMW−uPA	9.64 × 10^3^	3.98 × 10^−3^	4.13 × 10^−7^	6.33
	LMW−uPA	-*	-*	-*	-*
uPAapt−02	HMW−uPA	1.28 × 10^6^	2.77 × 10^−4^	2.17 × 10^−10^	1.49
	LMW−uPA	4.85 × 10^4^	1.43 × 10^−4^	2.95 × 10^−9^	0.03
uPAapt−02−R	HMW−uPA	8.98 × 10^5^	2.93 × 10^−4^	3.26 × 10^−10^	6.98
	LMW−uPA	6.55 × 10^4^	3.75 × 10^−4^	5.73 × 10^−9^	0.02
uPAapt−02−FR	HMW−uPA	2.42 × 10^5^	1.72 × 10^−4^	7.08 × 10^−10^	1.80
	LMW−uPA	2.47 × 10^4^	5.30 × 10^−4^	2.14 × 10^−8^	0.02
uPAapt−03	HMW−uPA	1.94 × 10^5^	6.50 × 10^−4^	3.34 × 10^−9^	0.02
	LMW−uPA	-*	-*	-*	-*
uPAapt−06	HMW−uPA	9.39 × 10^4^	9.99 × 10^−4^	1.06 × 10^−8^	3.55
	LMW−uPA	-*	-*	-*	-*
uPAapt−08	HMW−uPA	2.14 × 10^4^	8.53 × 10^−4^	4.00 × 10^−8^	0.20
	LMW−uPA	-*	-*	-*	-*
uPAapt−08−FR	HMW−uPA	7.77 × 10^2^	1.57 × 10^−4^	2.02 × 10^−7^	0.47
	LMW−uPA	-*	-*	-*	-*
uPAapt−21	HMW−uPA	1.58 × 10^5^	5.93 × 10^−4^	3.77 × 10^−9^	0.51
	LMW−uPA	4.74 × 10^4^	2.78 × 10^−3^	5.86 × 10^−8^	0.05
uPAapt−26	HMW−uPA	1.90 × 10^5^	2.46 × 10^−4^	1.30 × 10^−9^	0.64
	LMW−uPA	-*	-*	-*	-*
uPAapt−27	HMW−uPA	2.84 × 10^5^	1.31 × 10^−3^	4.59 × 10^−9^	0.11
	LMW−uPA	-*	-*	-*	-*
uPAapt−27−FR	HMW−uPA	1.93 × 10^5^	1.07 × 10^−3^	5.54 × 10^−9^	0.23
	LMW−uPA	-*	-*	-*	-*
Con	HMW−uPA	-*	-*	-*	-*
	LMW−uPA	-*	-*	-*	-*

Note: -*, no binding detected.

## References

[1] Mahmood N., Mihalcioiu C., Rabbani S.A. (2018). Multifaceted Role of the Urokinase-Type Plasminogen Activator (uPA) and Its Receptor (uPAR): Diagnostic, Prognostic, and Therapeutic Applications. Front. Oncol..

[2] Higazi A., Cohen R.L., Henkin J., Kniss D., Schwartz B.S., Cines D.B. (1995). Enhancement of the enzymatic activity of single-chain urokinase plasminogen activator by soluble urokinase receptor. J. Biol. Chem..

[3] Petersen L.C., Lund L.R., Nielsen L.S., Danø K., Skriver L. (1988). One-chain urokinase-type plasminogen activator from human sarcoma cells is a proenzyme with little or no intrinsic activity. J. Biol. Chem..

[4] Mekkawy A.H., Pourgholami M.H., Morris D.L. (2014). Involvement of urokinase-type plasminogen activator system in cancer: An overview. Med. Res. Rev..

[5] Masucci M.T., Minopoli M., Di Carluccio G., Motti M.L., Carriero M.V. (2022). Therapeutic Strategies Targeting Urokinase and Its Receptor in Cancer. Cancers.

[6] Dupont D.M., Madsen J.B., Kristensen T., Bodker J.S., Blouse G.E., Wind T., Andreasen P.A. (2009). Biochemical properties of plasminogen activator inhibitor-1. Front. Biosci..

[7] Li Santi A., Napolitano F., Montuori N., Ragno P. (2021). The Urokinase Receptor: A Multifunctional Receptor in Cancer Cell Biology. Therapeutic Implications. Int. J. Mol. Sci..

[8] Su S.-C., Lin C.-W., Yang W.-E., Fan W.-L., Yang S.-F. (2016). The urokinase-type plasminogen activator (uPA) system as a biomarker and therapeutic target in human malignancies. Expert Opin. Ther. Targets.

[9] Dass K., Ahmad A., Azmi A.S., Sarkar S.H., Sarkar F.H. (2008). Evolving role of uPA/uPAR system in human cancers. Cancer Treat. Rev..

[10] Setyono-Han B., Stürzebecher J., Schmalix W.A., Muehlenweg B., Sieuwerts A.M., Timmermans M., Magdolen V., Schmitt M., Klijn J.G.M., Foekens J.A. (2005). Suppression of rat breast cancer metastasis and reduction of primary tumour growth by the small synthetic urokinase inhibitor WX-UK1. Thromb. Haemost..

[11] Ossowski L., Russo-Payne H., Wilson E.L. (1991). Inhibition of urokinase-type plasminogen activator by antibodies: The effect on dissemination of a human tumor in the nude mouse. Cancer Res..

[12] Ma D., Gerard R.D., Li X.Y., Alizadeh H., Niederkorn J.Y. (1997). Inhibition of metastasis of intraocular melanomas by adenovirus-mediated gene transfer of plasminogen activator inhibitor type 1 (PAI-1) in an athymic mouse model. Blood.

[13] Muehlenweg B., Sperl S., Magdolen V., Schmitt M., Harbeck N. (2001). Interference with the urokinase plasminogen activator system: A promising therapy concept for solid tumours. Expert Opin. Biol. Ther..

[14] Schmitt M., Harbeck N., Brünner N., Jänicke F., Meisner C., Mühlenweg B., Jansen H., Dorn J., Nitz U., Kantelhardt E.J. (2011). Cancer therapy trials employing level-of-evidence-1 disease forecast cancer biomarkers uPA and its inhibitor PAI-1. Expert Rev. Mol. Diagn..

[15] Zhu F., Jia S., Xing G., Gao L., Zhang L., He F. (2001). cDNA transfection of amino-terminal fragment of urokinase efficiently inhibits cancer cell invasion and metastasis. DNA Cell Biol..

[16] Sato S., Kopitz C., Schmalix W.A., Muehlenweg B., Kessler H., Schmitt M., Krüger A., Magdolen V. (2002). High-affinity urokinase-derived cyclic peptides inhibiting urokinase/urokinase receptor-interaction: Effects on tumor growth and spread. FEBS Lett..

[17] Dupont D.M., Andersen L.M., Botkjaer K.A., Andreasen P.A. (2011). Nucleic acid aptamers against proteases. Curr. Med. Chem..

[18] Liu M., Zaman K., Fortenberry Y.M. (2021). Overview of the Therapeutic Potential of Aptamers Targeting Coagulation Factors. Int. J. Mol. Sci..

[19] Dupont D.M., Madsen J.B., Hartmann R.K., Tavitian B., Ducongé F., Kjems J., Andreasen P.A. (2010). Serum-stable RNA aptamers to urokinase-type plasminogen activator blocking receptor binding. RNA.

[20] Botkjaer K.A., Deryugina E.I., Dupont D.M., Gårdsvoll H., Bekes E.M., Thuesen C.K., Chen Z., Ploug M., Quigley J.P., Andreasen P.A. (2012). Targeting Tumor Cell Invasion and Dissemination In Vivo by an Aptamer That Inhibits Urokinase-type Plasminogen Activator through a Novel Multifunctional Mechanism. Mol. Cancer Res..

[21] Skrypina N.A., Savochkina L.P., Beabealashvilli R.S. (2004). In Vitro Selection of Single-Stranded DNA Aptamers that Bind Human Pro-Urokinase. Nucleosides Nucleotides Nucleic Acids.

[22] Zhou J., Rossi J. (2017). Aptamers as targeted therapeutics: Current potential and challenges. Nat. Rev. Drug Discov..

[23] Thevendran R., Citartan M. (2022). Assays to Estimate the Binding Affinity of Aptamers. Talanta.

[24] Dupont D.M., Thuesen C.K., Bøtkjær K.A., Behrens M.A., Dam K., Sørensen H.P., Pedersen J.S., Ploug M., Jensen J.K., Andreasen P.A. (2015). Protein-binding RNA aptamers affect molecular interactions distantly from their binding sites. PLoS ONE.

[25] Dupont D.M., Madsen J.B., Hartmann R.K., Tavitian B., Ducongé F., Kjems J., Andreasen P.A. (2015). Protein-binding RNA aptamers affect molecular interactions distantly from their binding sites. PLoS ONE.

[26] Schmidt C., Perbandt M., Klussmann S., Betzel C. (2020). Molecular characterization of a ghrelin-l-aptamer complex. J. Mol. Struct..

[27] Wang R.E., Wu H., Niu Y., Cai J. (2011). Improving the stability of aptamers by chemical modification. Curr. Med. Chem..

[28] Nobuhara M., Sakamaki M., Ohnishi H., Suzuki Y. (1981). A comparative study of high molecular weight urokinase and low molecular weight urokinase. J. Biochem..

[29] Gallagher S.R. (2006). One-dimensional SDS gel electrophoresis of proteins. Curr. Protoc. Mol. Biol..

[30] Wochner A., Cech B., Menger M., Erdmann V.A., Glökler J. (2007). Semi-automated selection of DNA aptamers using magnetic particle handling. Biotechniques.

[31] Garner M.M., Revzin A. (1981). A gel electrophoresis method for quantifying the binding of proteins to specific DNA regions: Application to components of the Escherichia coli lactose operon regulatory system. Nucleic Acids Res..

[32] Wochner A., Glökler J. (2007). Nonradioactive fluorescence microtiter plate assay monitoring aptamer selections. Biotechniques.

[33] Sass S., Stöcklein W.F.M., Klevesath A., Hurpin J., Menger M., Hille C. (2019). Binding affinity data of DNA aptamers for therapeutic anthracyclines from microscale thermophoresis and surface plasmon resonance spectroscopy. Analyst.

[34] Breitsprecher D., Schlinck N., Witte D., Duhr S., Baaske P., Schubert T. (2016). Aptamer Binding Studies Using MicroScale Thermophoresis. Methods Mol. Biol..

